# Navigating a learning journey in person-centered leadership: a grounded theory study

**DOI:** 10.1186/s12913-026-14798-3

**Published:** 2026-05-26

**Authors:** Jenny Wising, Charlotte Klinga, Ewa Carlsson Lalloo, Eric Carlström, Emmelie Barenfeld

**Affiliations:** 1 Department of Learning and Leadership for Health Professions, Institute of Health and Care Sciences, Sahlgrenska Academy, University of Gothenburg, Arvid Wallgrens Backe, Building 1, Box 457, Gothenburg, 405 30 Sweden; 2 Gothenburg Centre for Person-Centred Care (GPCC), University of Gothenburg, Gothenburg, Sweden; 3 Departement of Learning, Informatics, Management and Ethics, Karolinska Institutet, Stockholm, Sweden; 4 Academic Primary Healthcare Centre, Stockholm Health Care Services, Stockholm, Sweden; 5 Faculty of Caring Science, Work Life and Social Welfare, University of Borås, Borås, Sweden; 6 Department of Health and Rehabilitation, Institute of Neuroscience and Physiology, Sahlgrenska Academy, University of Gothenburg, Gothenburg, Sweden

**Keywords:** Person-centred, Leadership, Person-centred leadership, Education, Leadership education, Constructivist grounded theory, Leadership development, Integrated care, Person-centred care, Patient-centred care

## Abstract

**Background:**

Global challenges call for more integrated and person-centered healthcare. Effective leadership is essential in enabling this shift, and leadership education plays an important supporting role. In Sweden, a leadership program focusing on person-centered leadership has been developed to facilitate the transition to person-centered and integrated care (PC-IC). However, despite increasing demand, such programs remain largely unexplored from a learning perspective. This study aims to explore learning processes among leaders participating in a leadership program designed to support the transition toward person-centered and integrated care.

**Methods:**

A constructivist grounded theory approach was applied in data collection and analysis. Individual interviews were conducted with eleven leaders from diverse organizations and levels across Sweden who participated in the leadership program in either 2022 or 2023. Data from participants’ final graduation presentations on lessons learned were theoretically sampled.

**Results:**

The program allowed leaders to adapt the educational content to professional contexts, personalizing their learning experience. This aspect is captured in the core category *navigating a person-centered learning journey*. Three parallel categories capturing processes (*orienting on a person-centered inner journey*,* exploring person-centeredness with others*, and *operationalizing person-centeredness in practice*), together with the contextual category *challenged in different arenas* provide insights into facilitators and barriers of learning person-centered leadership.

**Conclusions:**

Taken together, the findings highlight that learning person-centered leadership is best understood as an ongoing, iterative journey. Three interconnected processes uniquely influenced this journey, through which leaders actively shaped and adapted their learning via reflection, shared exploration, and practical application. These processes were shaped by contextual conditions that both facilitated and constrained engagement, providing insights for designing leadership programs supporting PC-IC.

**Supplementary Information:**

The online version contains supplementary material available at 10.1186/s12913-026-14798-3.

## Background

Healthcare leaders must navigate complex systems while adapting to an evolving landscape that includes shifting roles and expectations [1]. Patients increasingly seek greater involvement in how their care is organized, accessed, and delivered [2, 3]. Moreover, higher levels of patient involvement are associated with improved quality of care and enhanced patient safety [4, 5]. Consequently, leaders must adopt innovative approaches and prioritize patient involvement. The World Health Organization (WHO) has recognized this need for transformation and advocates integrated, people-centered healthcare [6]. This transformation is evident in policies supporting this shift, such as the Swedish reform Person-Centered and Integrated Care (PC-IC) [7]. Translating person-centered principles into actions involves building and nurturing healthy relationships among all stakeholders involved in healthcare provision, including patients, their significant others, and healthcare providers [8]. By leading in a person-centered way, leaders can play a vital role in driving this transformation [9–11].

Descriptions of person-centered leadership vary, but in summary, it is a complex, relationship-focused, and adaptable approach [12, 13]. In this study, person-centered leadership is defined as integrating person-centered principles into both one’s being and one’s doing [11]. It involves consistently manifesting these principles and adopting them at a deeper, internal level so that they naturally influence attitudes, perspectives, and behaviors [11, 14]. Specifically, it requires that leaders acknowledge and respect a person’s unique needs, preferences, and capabilities, upholding their right to autonomy, and endeavoring to achieve mutual understanding [8, 9]. From this perspective, leadership for PC-IC extends beyond interactions with patients and staff, encompassing collaborative partnerships with co-workers as a key element in translating person-centered principles into leadership practice [15].

There is an advocated need for educational programs that promote person-centered leadership [10, 16]. Education has demonstrated effectiveness in clinical practice, both in improving care outcomes [17–19] and in shaping leadership practices [18, 20], and is widely applied as a strategy for implementing person-centered care [21]. However, this approach has also been subject to criticism. It has been argued that educational initiatives alone are insufficient to effect change in complex interventions such as person-centered care [22]. While previous studies have examined person-centered outcomes of educational programs aimed at developing leadership capabilities among healthcare staff [18, 20], few person-centered education programs have specifically targeted individuals in formal leadership positions. Bradd et al. [20] evaluated a leadership program grounded in transformational leadership and reported that it supported changes in workplace culture and facilitated the implementation of person-centered practices. Similarly, a study examining a person-centered educational initiative for leaders identified a shift toward collaborative approaches to achieve person-centered change, rather than perceiving person-centeredness as imposed [18]. Other research has explored the development of person-centered leadership through leaders´ direct engagement in the research process [12]. To our knowledge, no studies have specifically focused on how leaders learn person-centered leadership within the context of a leadership program.

Understanding the core dimensions of competence development such as developing person-centered leadership, requires attention to the factors that shape enabling and constraining conditions for learning and growth [23]. Leadership development is widely conceptualized as a process-oriented phenomenon, characterized by dynamic and interrelated learning processes that evolve through experience over time [24]. By exploring leaders’ learning processes within a leadership program, this study aims to advance understanding of how person-centered leadership is learned and how leadership programs can be designed to better support the transition toward PC-IC. Although person-centered principles have been incorporated into curricula across diverse conceptual frameworks and settings [18, 25, 26], their systematic application within leadership programs remains limited. The present program was informed by the framework developed at the Gothenburg Center for Person-centered Care (GPCC), which operationalizes person-centered principles through key routines, including eliciting the patient narrative, working in partnership through shared decision-making, and safeguarding the partnership through documentation [27]. While these GPCC’s routines have demonstrated effectiveness at both an individual and organizational level [10], they have not previously been examined within the context of a leadership education.

## Aim

This study aims to explore learning processes among leaders participating in a leadership program designed to support the transition toward person-centered and integrated care.

## Methods

### Study design

This qualitative study employed a constructivist grounded theory (con-GT) approach inspired by Charmaz [28]. Con-GT is well suited for identifying processes and actions [28, 29], and was therefore considered appropriate for exploring how leaders learn within a leadership program focused on person-centered leadership. The study was designed with input from a diverse group of patients, family representatives, and leaders in healthcare to ensure a well-rounded and inclusive perspective. This study was reported in accordance with the Consolidated Criteria for Reporting Qualitative Research (COREQ) [30].

### Study context

This study is part of a larger project evaluating a leadership program provided to leaders in Sweden [13]. The program has been developed and organized collaboratively with representatives of the Swedish Association of Health Professionals (SAPH) and researchers from GPCC. SAPH is the trade union for nurses, midwives, biomedical analysts, and radiology nurses.

The program was designed to support the development, enactment, and implementation of person-centered leadership and practice. It corresponded to 7.5 higher education credits and was delivered over a six months period through six modules structured around the three key routines of the GPCC framework [13]. The program was not anchored in a specific leadership theory, rather it emphasized relational dimensions of leadership, such as dialogue and collaboration. These elements reflected recurring themes across the broader leadership literature, including transformational [31] and relational leadership [32], and align closely with person-centered principles [27].

**Fig. 1:**
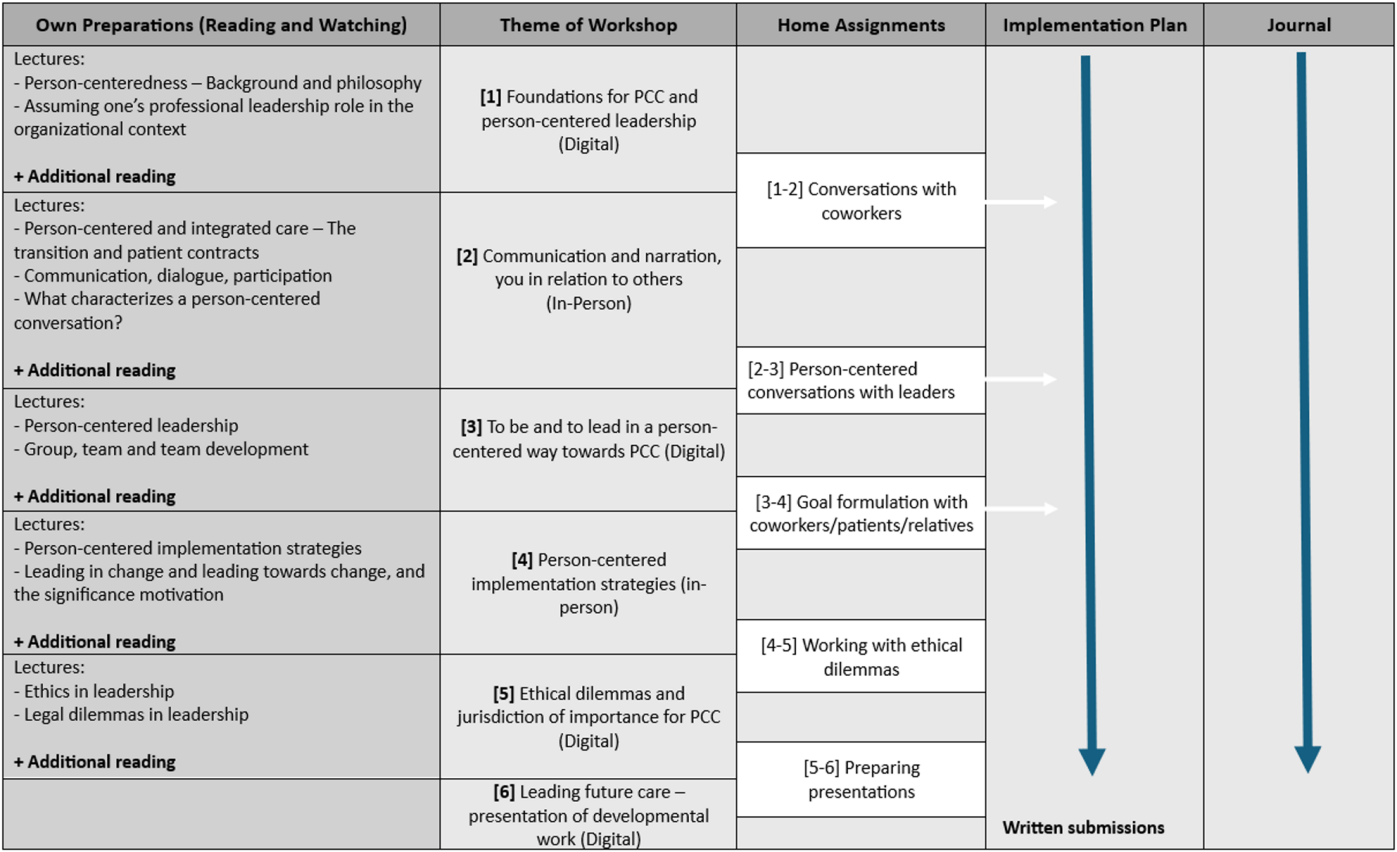
Overview of the educational curriculum

A blended learning environment was used, combining in-person and digital workshops with practice-based learning embedded in participants’ own organizational contexts between workshops. The program included diverse learning activities as shown in Fig. 1. In addition, participants developed an implementation plan and engaged in structured reflective practices, including maintaining a reflective journal throughout the program.

Two editions of the leadership program, year 2022 and 2023, were included in this study. The 2023 program contained two in-person meetings rather than one, and modules 3 and 4 were delivered in reversed order. Otherwise, the content and pedagogical approach remained the same. For further details see Lood et al. [13].

### Participants and sampling

Participants were recruited on a rolling basis via email by CK and JW following the 2022 and 2023 editions of the leadership program. An additional inclusion criterion was that applicants either had explicitly referred to PC-IC in their application to the leadership program or in an assignment completed during the program or had professional experience within a primary care context. This criterion was applied to ensure that participants possessed familiarity with the Swedish government-supported initiative PC-IC [7, 33], in which primary care functions as the central coordinating hub [33].

Leaders who consented to participate in the study and met the inclusion criteria were selected using purposefully sampling, guided by predefined criteria established at the outset of the study. The sampling strategy aimed to ensure diversity in participants’ length of leadership experience and scope of managerial responsibility as indicated by the number of co-workers, as well as variation in the patient populations served across organizational settings.

In total thirteen leaders were asked to participate, and all but two agreed. The reasons for declining participation were personal and involved a lack of time to participate in research. Informed and written consent was obtained from all participants. The eleven participants were all women, and all were registered nurses currently in leadership positions. Their age ranged from 31 to 59 years, with a median age of 51 years. The majority (*n* = 7) were unit managers; others were operations managers and an innovation manager. The number of co-workers ranged from 0 to 75 co-workers per manager (mean 32). Further information about the participants’ characteristics is presented in Table 1.

**Table 1:** Participants’ characteristics

Participant	Years as a leader	Organization
Leader 1	3–5	Social Services
Leader 2	6–10	Social Services
Leader 3	6–10	Primary Care
Leader 4	3–5	Social Services
Leader 5	3–5	Municipal Care
Leader 6	1–2	Primary Care
Leader 7	3–5	Primary Care
Leader 8	6–10	Hospital Care
Leader 9	1–2	Primary Care
Leader 10	3–5	Primary Care
Leader 11	6–10	Primary Care

### Data collection and analysis

Data collection and data analysis occurred concurrently [28]. Interviews were conducted within four months after program completion. Those in the 2022 cohort (*n* = 6) were interviewed by CK during January and February 2023, while those in the 2023 cohort (*n* = 5) were interviewed by JW during October and December 2023. The interviews were conducted using face-to-face video conferencing software and their duration varied between 36 and 58 min (mean 50 min).

All interviews started with an open question to capture the start of the learning process “’Can you please tell me what made you apply to the leadership program?’. Follow-up questions and exploration of pre-identified question areas followed the opening question. The interview guide is available in Additional file 1. New question areas were introduced when interviewing participants in 2023. The refinement of question areas during the study is integral to theoretical sampling, and is intended to enhance the comprehension of emerging categories [34, 35]. In the process of theoretical sampling, the audio from the three-minute video presentations delivered by the interviewed participants at the time of their graduation were incorporated into the analysis.

Audio recordings of the interviews and the presentations were transcribed verbatim. All the transcripts were imported into ATLAS.ti 23 [36], as also was text from the PowerPoint slides used in the graduation presentations. ATLAS.ti 23 served as a tool to structure and facilitate the analysis.

The first author performed the main data analysis, beginning with line-by-line coding in which a code was assigned to every line of dialogue [28]. During this coding process, there was constant comparison between and within the transcripts and various codes. Once robust analytic directions had been identified through initial line-by-line coding, the next step was focused coding during which larger segments of data were synthesized and conceptualized to further advance the comparative process [28]. By revisiting the initial codes, going back to the text, exploring memos typed during the initial analysis, and engaging in discussions with the last author, codes were clustered to identify the initial central categories. Finally, data and focused codes were theorized to create an analytical narrative that was coherent and assisted in specifying possible relationships between categories [28]. The analysis continued until the final draft was formulated to present a theory that was discussed by all authors. When the analyses did not uncover any new findings regarding the emerging theory after about nine interviews, it was concluded that saturation had been reached even though eleven interviews in total were conducted.

## Results

**Fig. 2:**
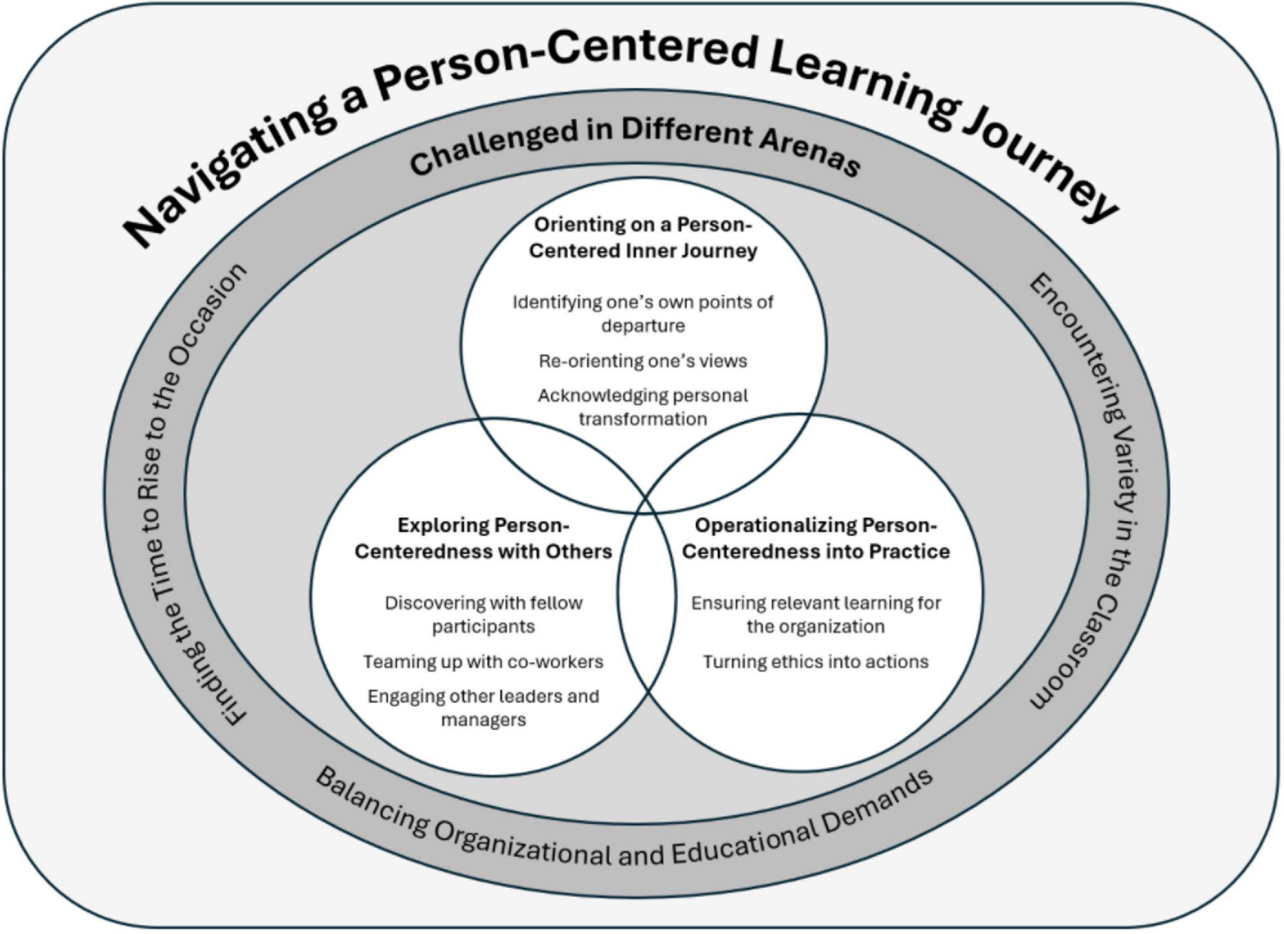
Visualization of the leaders’ learning processes and how they interact and are influenced by contextual learning conditions

The analysis demonstrated that leaders learned person-centered leadership through three interrelated learning processes: o*rienting on a person-centered inner journey*,* exploring person-centeredness with others*, and *operationalizing person-centeredness into practice*. These processes shaped how, when, and with whom learning unfolded during leaders´ participation in the program. Although analytically presented as distinct categories, the learning processes were iterative in nature; orienting, exploring, and operationalizing occurred in a dynamic interplay with learning continuing to evolve as leaders progressively integrated person-centered leadership into their professional practice. Leaders’ learning experiences were further influenced by contextual conditions within their workplaces and during program workshops, captured in the contextual category *challenged in different arenas*. In addition, leaders actively shaped their learning during participation in relation to their personal values, experiences, and professional identities. Collectively leaders´ experiences were rendered as an ongoing learning process, characterized as *navigating a person-centered learning journey* that extended beyond the formal leadership program. This overarching process constituted the core category. A visual representation of the learning journey and the interrelationship among categories is presented in Fig. 2.

### Orienting on a person-centered inner journey

*Orienting on a person-centered inner journey* captures how leaders engaged in ongoing self-development while learning person-centered leadership. *Orienting* in this context refers to how the leaders experienced positioning and aligning themselves in regard to person-centeredness. The *inner journey* represents their experience of how continuously exploring and mapping their personal thoughts contributed to learning person-centered leadership and fostered self-growth. Three subcategories of this inner journey were identified, namely *identifying one’s own points of departure*,* re-orienting one’s view*, and *acknowledging personal transformation.*

#### Identifying one’s own points of departure

*Identifying one’s own points of departure* involved each of the leaders’ identifying their own perspective on person-centeredness when they were admitted to the leadership program. This necessitated introspection and self-assessment as they reflected on their own preunderstandings and views on person-centered leadership. A wide range of experiences, such as previous healthcare experience, education, and positions of trust, shaped their views and were among the reasons they enrolled in the leadership program. These reasons included wanting to learn more about leadership, wanting to improve their organization, curiosity about person-centeredness, and a desire for personal development:

*And I think that was something we also discussed in our study group*,* that we were. . . We were really in different places regarding it [person-centeredness]. Some were there to get help with their organization and some*,* like me*,* were more interested in personal development*. (Leader 2)

#### Re-orienting one’s view

The leaders found that they had to *re-orient their view* of person-centeredness as they gained new insights and perspectives in the course of the program. These new perspectives changed and deepened their original understanding of person-centered leadership. For example, leaders chose to relisten to digital lectures because they found new meaning in them after reflecting on the latest information and knowledge derived from the program. Such re-orienting helped them to figure out ways to improve person-centered leadership in the future. A recurring insight was that person-centeredness does not relate solely to encounters with individual patients but is applicable in several parts of the organization:

*There were a lot of question marks about this whole thing with person-centered leadership. What does it really mean? I read about it*,* and it said something like “you should listen actively*,* be fully present. . . ” Yes*,* yes*,* but I’ve read that many times. What does it really mean? And when I really understood it*,* then I felt “Yes! That’s when the penny dropped”.* (Leader 7)

#### Acknowledging personal transformation

*Acknowledging personal transformation* entails recognizing or accepting changes or developments within oneself. It involved being aware of and validating the process of personal growth and change, influenced by both introspection and external feedback from others. The most significant changes occurred internally. By acknowledging and using their existing strengths, the leaders improved their leadership skills. They recognized new approaches to communication with co-workers, such as becoming a better listener and showing an increased desire to engage with patient representatives. They also found that the benefits of the leadership program extended beyond their current workplace, as the personal growth could be applied in any organizational setting:

*I think I may have had preconceived ideas about what a good leader should be and how they should act*,* and I’ve relied on those when stepping into this role. You’re supposed to be available*,* to be there. And I am very available in my organization. But now [after participating in the program] I feel that I have the confidence to step back and not be physically present so that I can be mentally present in a different way when I am there.* (Leader 9)

### Exploring person-centeredness with others

*Exploring person-centeredness with others* captures how leaders learned collectively through interactions with fellow course participants, co-workers, and other leaders and managers within their organization. This contributed to different perspectives. The process is further detailed in the following subcategories: *discovering with fellow participants*,* teaming up with co-workers*, and *engaging other leaders and managers.*

#### Discovering with fellow participants

The leaders’ experiences on the collaborative journey of *discovery with fellow participants* indicated that discussions, idea sharing, and personal reflections were essential for their gaining new insights into the meaning and application of person-centered leadership. A significant catalyst for discovery was the diverse experiences of the leaders, stemming from their backgrounds in different organizations, geographical locations, and leadership positions. These differences became a source of inspiration, shifting the focus to exploring leadership styles rather than focusing solely on person-centered care in their own specific context. Although the manager’s role is portrayed as lonely, the leaders felt they could be vulnerable with fellow participants and experienced camaraderie. The resulting safe and supportive environment encouraged exploration and discovery. The camaraderie among participants also reinforced a sense of personal responsibility for their learning:

*It was like you share and talk with each other [fellow participating leaders] about how we can work more person-centered*,* both as leaders and in how we provide more person-centered care and services in our organizations during the transition. It was so rewarding to get so many different perspectives*. (Leader 4)

#### Teaming up with co-workers

Leaders found it vital to *team up with co-workers* during the leadership program, which helped them to recognize the value of collaboration and the importance of working together toward a common goal. They also spoke of using the program to build a shared understanding of person-centered leadership and to define the expectations of a person-centered leader and person-centered care in the workplace. By integrating lessons from the program into workplace meetings, they educated their co-workers and sparked conversations about person-centeredness. Through lectures and discussions, the leaders and their co-workers learned the value of sharing responsibilities, pooling resources, and using each other’s strengths. This teamwork and shared decision-making approach carried over into the leaders’ assignments, where they relied on their co-workers for input and suggestions to identify areas for improvement:

*It’s pretty easy*,* I think*,* as a manager today to go into a meeting saying*,* “This is what I want to raise with you; this is what I want us to talk about*,*” or “This is the purpose of our conversation.” But the other person might have a different agenda. So I think that’s something new I’ve taken with me—the idea of setting an agenda together in these meetings. Even before starting the conversation*,* asking*,* “What’s important for you that we talk about today?”* (Leader 11)

#### Engaging other leaders and managers

By seeking collaboration and input, the leaders proactively *engaged other leaders and managers* at different organizational levels to learn person-centered leadership. The enthusiasm for person-centered leadership they had gained during the leadership program motivated them to build a supportive network focused on a more person-centered approach. They also tested their knowledge by encouraging other managers and leaders to reflect on and discuss person-centered leadership and care. Despite facing resistance, they persevered in their efforts to involve others in the transition to increased person-centered leadership:

*And then I wanted to share this video [a lecture about person-centered leadership] with all the other managers at the hospital*,* so I sent it to our management team like this: “I would like to share a video” And I don’t understand why it’s so difficult for. . . the majority of managers to embrace things like this. . . But now*,* in two weeks*,* it’s time!* (Leader 8)

### Operationalizing person-centeredness into practice

O*perationalizing person-centeredness into practice* entails how the leaders translated theoretical insights into action within their organization by integrating classroom learning with practical experience. In doing so, they solidified their understanding through applying theoretical concepts in real-world situations. The process is elaborated in the two subcategories: *ensuring relevant learning for the organization* and *turning ethics into actions.*

#### Ensuring relevant learning for the organization

*Ensuring relevant learning for the organization* refers to the leader’s creating educational opportunities that directly addressed and were adapted to the needs of their organizations. They used identified organizational needs to shape their assignments during the leadership program, and the program was also integrated into existing projects. The program also inspired the leaders to identify areas for improvement in their organizations. The graduation presentations addressed a variety of topics, such as creating new procedures for admission interviews, including patient representatives in improving, evaluating and developing care, and enhancing team collaboration. Focusing on aspects of the program that directly benefited their organization and prioritizing a clear and immediate connection to their everyday work life meant that the leaders were excited to keep on driving person-centered changes post-graduation:

*If I’m going to do this [participate in the program]*,* it should be useful. There’s no point in me just making something up; instead*,* we’re doing this based on the fact that it aligns closely with the work we’re already doing.* (Leader 2)

#### Turning ethics into actions

*Turning ethics into actions* reflects the leader’s experience of making theoretical ideas more concrete in order to better understand person-centered practice. They felt that the leadership program helped them describe previously abstract concepts and identify existing person-centered aspects in their organizations. The program also provided practical tools for implementing a person-centered approach, including clear, real-world examples during lectures. Additionally, the implementation plan crafted during the program played a pivotal role in articulating clear objectives aimed at promoting person-centered leadership:

*I selected some video clips*,* where they had conducted interviews with patients*,* and I showed those video clips I found on YouTube. I then mixed in some philosophical terms from the literature. I aimed to spark discussions around questions like*,* “How can we. . . ?” and “What does a person-centered approach mean for us?” Because there are also incredibly different variations of that concept when you’re in a work group. So I tried to present it in a way that makes the abstract concept more tangible.* (Leader 1)

### Challenged in different arenas

Being *challenged in different arenas* highlights how environmental factors shaped and influenced the leader’s learning journey. Being challenged emphasizes how these factors fostered problem-solving, adaptation, and the willingness to step outside one’s comfort zone for personal growth. It also illustrates how leaders recognized contextual strengths in the face of adversity. The d*ifferent arenas* were the main settings of their learning: the classroom and their organization. Three subcategories elaborate on this: *encountering variety in the classroom*,* balancing organizational and educational demands*, and *finding time to rise to the occasion.*

#### Encountering variety in the classroom

The leaders described how being immersed in a dynamic educational setting characterized by various teaching methods, tools, and technologies impacted their learning about person-centered leadership. While there was overall appreciation for the program’s flexible format, which allowed them to tailor their education to their individual needs, some felt a need for more guidance and clearer instructions. The combination of individual and group-based activities, lectures, and practical assignments was experienced as creating an engaging learning atmosphere. Small group and whole-class meetings created trust and encouraged the exchange of diverse perspectives. However, it became clear that when small groups did not function well because of dropouts or lack of engagement, the leaders reported relying more on support from people outside the leadership program.

The leaders also reflected on the difference between learning about person-centered leadership digital versus in physical settings and appreciated the value of real-time discussions and face-to-face interaction. Digital meetings and asynchronous communication were deemed essential to facilitate participation due to their efficiency and convenience; however, they also led to feelings of detachment and passivity. Encountering classroom variety contributed to the three categories of learning processes identified above by creating a dynamic and engaging learning environment that stimulated their interest in the program:

*I preferred that it was as varied as it was*,* so it wouldn’t just be. . . not just basic group work and not only digital lectures or home assignments. I think the mix is what made it enjoyable between the meetings*. (Leader 3)

#### Balancing organizational and educational demands

The leaders were aware of having to balance both organizational and educational demands if they were to meet the expectations and responsibilities of their workplace and of the leadership program. They recognized a shift toward PC-IC and engaged in forums on the topic outside the program, viewing organizational changes as central to their leadership role. As they saw how the program could support this transformation, their sense of responsibility and pressure to use what they were learning increased. However, fully engaging with the program required time and focus, reducing their presence at work, and sometimes forcing them to choose between their organizational duties and engagement in the program. This balancing act drove the three process-related categories identified by emphasizing how the program could benefit the organization. Yet, it also generated unease, which may have affected their focus during workshops:

*And that anxiety [being away from the workplace]*,* to be completely honest*,* was a bit tough. It’s like something always manages to happen when you’re away*,* and then you’re just sitting there. We tried. . . tried to turn off the phone*,* but as soon as there’s a break*,* you check it*,* and someone calls*,* and there’s something going on and. . . You just can’t fully disconnect from work.* (Leader 10)

#### Finding the time to rise to the occasion

The leaders emphasized that their concerns were not just about having time per se, but about *finding the time to rise to the occasion* when learning and practicing person-centered leadership. They emphasized the importance of allocating time to learn person-centered leadership in a hectic workplace, highlighting several key reasons: time is crucial for reflection and is necessary to foster sustainable change and promote person-centered approaches in both care and leadership. The leaders supported their co-workers in stepping up to the challenge. By engaging co-workers who were receptive to change, they aimed to harness their enthusiasm to gradually inspire others.

Leaders also reflected on the timing of the different parts of the leadership program. The fact that it was spread over six months made it easier for them to incorporate it in their schedules, but the long intervals between meetings sometimes disrupted continuity. Modules were released one by one, which kept them focused but limited their preparation time. Scheduling occasionally conflicted with a demanding workload, which made it more challenging to allocate time for learning about person-centered leadership. The leader’s commitment to finding time to rise to the occasion was essential to facilitate the three process-related categories, particularly the act of reflection:

*Because now I feel like I’ve been through an extremely intense period*,* and I can sense. . . I just feel like*,* “no*,* now I need time for reflection” when you feel like “I haven’t had time to reflect*,* and it’s not going well.” And that’s something the course really highlights—how important it actually is*,* and that you need to set aside time for it. But how incredibly easy it is to take that time away in favor of other things that just have to get done.* (Leader 11)

## Discussion

To the best of our knowledge, this is the first study to explore how leaders learn while participating in a leadership program focused on person-centered leadership. The primary finding of this study is how leaders *navigated a person-centered learning journey*, actively shaping and adapting their learning processes. This journey involved three interrelated processes: *orienting on a person-centered inner journey*,* exploring person-centeredness with others*, and *operationalizing person-centeredness in practice.* The discussion elaborates on these findings in relation to the contextual conditions of being *challenged in different arenas* and draws conclusions that point to recommendations for the design of future programs in person-centered leadership.

When the leaders *oriented on a person-centered inner journey*, they drew on their lived experience and self-narratives for introspection and self-reflection. A key finding was that this process deepened their understanding of person-centeredness and fostered personal transformation. This finding also resonates with descriptions of transformative learning, particularly in terms of shifts in frames of reference [37]. Ongoing reflection is also essential for integrating person-centered principles, which require adaptation to unique contexts [9]. Our findings suggest that the program created conditions that supported personal reflection and inner growth, as seen in previous person-centered educational studies [18, 26]. Furthermore, the result indicates that this personal growth may remain applicable across organizational settings, even when leaders change workplaces.

As shown in previous research incorporating variety to support diverse learning needs is encouraged to facilitate learning about person-centeredness [38, 39]. Aligning with this body of research, the present study shows that *encountering variety in the classroom* created an engaging learning atmosphere. This variety also included digital meetings and asynchronous communication, and our findings extend existing understandings of how blended learning environments influence learning person-centred leadership. In example, recorded lectures enabled the leaders to revisit content at their own pace, supporting ongoing learning and *re-orienting one’s views* on person-centeredness. Digital meetings also facilitated attendance as they were time-efficient, making it easier to *balance educational and organizational demands*. Digital formats are also recognized as cost and time-efficient [40]. Despite the potential benefits of varied and flexible learning environments, integrating digital elements in education presents well-known challenges [41], as reflected in leaders’ description of the risk of feeling detached and passive during digital meetings.

To learn person-centered leadership, the leaders needed to *explore person-centeredness with others* such as fellow participants, co-workers, and other leaders and managers within their organization. Previous research shows that feedback helps reduce feelings of loneliness, boosts motivation, and encourages the development of leadership qualities [42]. In our study, the leaders created a feedback system among themselves. A key finding was how the leaders learned person-centered leadership together with leaders from diverse backgrounds during the program. Creating spaces for such interaction is essential to promoting collaboration [43].

Leaders collaborating across organizational boundaries while learning person-centeredness benefit not only in terms of the educational context but also in terms of broader inter-organizational collaboration. Such interactions are integral to developing a person-centered and integrated healthcare system [9, 10]. There are, however, often challenges to collaborative interaction in inter-organizational environments [11, 12]. For example, leaders may focus on safeguarding their own interests and may not fully comprehend the operations of their partnering organizations [44]. But during the program, leaders exchanged experiences and realized that, despite their diverse backgrounds, they faced similar challenges in transitioning to PC-IC. Research has shown that aligning healthcare staff around shared values enhances collaboration and motivates them to work toward common goals [45, 46]. Learning person-centered principles can thus unite leaders around a common philosophy, bridge organizational differences, enhance mutual understanding, and support inclusive care for patients, all elements that support the transition to PC-IC [47]. This knowledge highlights how group composition and participant selection may influence learning processes within leadership programs.

Although working with patients was part of the curriculum, and despite leaders showing increased interest in involving patient representatives, in the interviews none of them mentioned learning during the leadership program together with patients. Co-creation with patients is essential in person-centered care [48], and these principles were likely considered so fundamental to a person-centered approach that they were not explicitly included in the learning outcomes of the leadership program [13]. This study, therefore, identified the need to clarify and amplify this aspect within the content of the leadership program.

In *operationalizing person-centeredness in practice*, the leaders applied insights and homework assignments from the program directly within their organizations. A key finding was that leaders strengthened their theoretical understanding by applying practical concepts in their workplace, which encouraged ongoing learning after graduation. While integrating person-centered principles into a curriculum does not guarantee their practice [46], practical assignments in this leadership program may have provided a bridge from knowledge to action, aiming to support sustainable change [13]. Leaders’ commitment to continuing implementing person-centered changes after graduation indicates that the practical assignments were effective, in line with previous research [42]. This alignment supports the overall goal of person-centered education, which is continuous learning [46].

The leaders attending the program were aware of the transition toward PC-IC. When *balancing educational and organizational demands*, recognizing the leadership program’s contribution to organizational change increased their sense of responsibility and engagement in learning. Promoting person-centeredness requires a cultural change [18, 46], and so education programs must strategically target such shifts [46]. While implementing person-centered structural changes can reduce resistance over time, transforming organizational culture remains challenging [49]. This study shows that leaders remained committed despite facing resistance. They strove to *find time to rise to the occasion* and planned to be persistent in driving changes in person-centered practice. Encouraging continuous development and practical application may support lasting organizational change. At a broader level, this approach can expand PC-IC in healthcare, so meeting the demands for patient involvement [48].

As a broader reflection, the leadership program’s integration of GPCC’s three key routines—narrative, partnership, and documentation [13]—offers a conceptual framework for structuring learning in person-centered leadership. Although the leaders did not directly mention these routines, their influence was clearly discernible. They used their self-narratives [50] to *orient on a person-centered inner journey*, aiding in structuring and comprehending existing and new knowledge. In *exploring person-centeredness with others*, it is evident how the leaders partnered [9] and co-constructed their learning with others, and fostered these partnerships through shared narratives. Finally, in o*perationalizing person-centeredness into practice*, the leaders’ experience of concretizing and integrating the program within the organization resembles the act of documentation [27], enhancing transparency and ensuring continuity. Research indicates that translating person-centered frameworks into practice can be complex [51]. However, our results suggest that integrating GPCC’s routines may support how person-centered leadership is learned and enacted in practice.

## Strengths and limitations

In constructivist grounded theory preunderstanding strengthens the conceptualization of data [28], but may limit theoretical sensitivity due to the researchers’ reliance on these preunderstandings [52]. Their own personal histories, perspectives, values, and interests influence the questions asked and the interpretation of the data [28]. These issues were addressed by acknowledging potential partialities during discussion and by adhering to COREQ guidelines [30].

The first author (JW) had no contact with the leaders before the interviews. The second author (CK), who developed and managed the program, participated as a lecturer during the leadership program. While this may have resulted in more personal conversations during the interviews, it also raises ethical considerations [53] as, for example, it may have discouraged negative feedback. Despite this, the interviews still reveal both facilitators and barriers when it comes to how the program contributed to learning person-centered leadership. A further limitation was that the interviews were conducted by video meetings, which is often seen as less effective than in-person interviewing for obtaining rich descriptions [54].

The data analysis was primarily conducted by first author JW, with support from the last author (EB), who also contributed to the development and management of the program. EB’s involvement might have influenced the interpretation of the results of the program through her preconceptions. EB was, however, experienced in the methodology, whereas JW was a novice. EB’s guidance to JW strengthened the theoretical sensitivity [52], and so can be said to have enhanced both the credibility and usefulness of this study [28]. To gain further perspectives on the theory conceptualization, the third author (ECL) and fourth author (EC), together with a reference group, were engaged in discussing the emerging theory.

Another limitation of this study were that all the participants were female and thus there was a lack of male representation, which is consistent with the dominance of female healthcare managers in Sweden [55]. There was, however, male representation (EC) in the research group.

When considering this study’s resonance [28], it is important to note that the participants were self-selected, motivated leaders interested in person-centeredness, and so did not represent leaders unfamiliar with or uninterested in this practice, or those who withdrew from the program. Also, PC-IC centers around primary care [33], which was included as a sampling criterion. However, this criterion increased the homogeneity of the included organizations, and all participants were nurses, limiting professional diversity. There are thus opportunities for refining the training content and structure, as well as a need for studies that systematically examine the benefits of including broader perspectives. Additionally, this theory is tied to a specific context, which readers should consider when interpreting the results. Future studies could apply the theory in varied settings, gathering additional data to refine and expand the categories based on new evidence.

## Conclusion

This study advances a process-oriented understanding of how person-centered leadership is learned following a leadership program aiming to support the transition toward PC-IC. Overall, learning person-centered leadership can be understood as an ongoing and iterative journey, shaped by three processes, each contributing uniquely to leadership development. Learning unfolded through the interplay of introspection and reflection, shared exploration with a variety of partners (e.g. co-workers, and leaders), and practical application in the workplace, highlighting the importance of leadership programs that intentionally support all three to reinforce and deepen learning. Importantly, these processes were influenced by contextual conditions, with differences in leaders’ circumstances influencing the scope and depth of their learning. Varied learning activities, blended learning environments, and opportunities for collaboration could both facilitate and constrain learning. These insights can be used to inform the design of leadership programs in person-centered leadership.

## Electronic Supplementary Material

Below is the link to the electronic supplementary material.

Supplementary Material 1

## Data Availability

No datasets were generated or analysed during the current study.

## Declarations

### Author information

JW: Registered nurse, specialist nurse in cardiovascular care and intensive care, Ph.D. student, female. CK: Social worker, licensed healthcare counselor, M.D, female. ECL: Registered nurse, senior lecturer, Ph.D., female. EC: Registered nurse, specialist nurse in paramedic care, professor in nursing science, associate professor in public administration, former manager in several healthcare organizations, male. EB: Occupational therapist, associate professor in occupational therapy, female.

## Abbreviations

GPCC: Gothenburg Center for Person-Centered Care
PC-IC: Person-centered and integrated care
