# Supplementary material for: Navigating a learning journey in person-centered leadership: a grounded theory study
Source: BMC Health Serv Res. 2026 May 26;26:759. doi: 10.1186/s12913-026-14798-3 (PMC13214110; doi:10.1186/s12913-026-14798-3)
Supplement: Supplementary file 1 — Supplementary Material 1 [file 12913_2026_14798_MOESM1_ESM.docx]

# **Opening Question**

Can you please tell me what made you apply to the leadership program?

## **Question areas**

**Overall**

Person-centeredness before and after the program

Leadership before and after the program

**Pedagogical setup**

Events during the leadership program of specific significance for personal development/ learning? (Within the organization, the program and other contexts)

Structure and content of the program:

- Module 1-6
- Assignments included during the program
- Peer-learning and individual learning
- Digital meetings and face-to-face interactions

Barriers and facilitators for learning

**Question areas introduced after interview 7**

Experiences of theoretical and concrete parts of the program

Experiences of creating freely and receiving guidance

Experiences of similarities and differences when learning with other leaders

**Additional questions**

If you were responsible for the course, how would you have wished to develop it further?

Is there anything else you would like to share that I haven’t asked about?

Is it okay if I come back if I think of somehing I missed asking about?
